# Comparative Analysis of Double-Stage Interfaces for Electromagnetic Energy Harvesters Generating Impulsive Voltages

**DOI:** 10.3390/s26196213

**Published:** 2026-09-30

**Authors:** Alessandro Lo Schiavo

**Affiliations:** Department of Engineering, Università degli Studi della Campania “Luigi Vanvitelli”, Via Roma 29, 81031 Aversa, CE, Italy; alessandro.loschiavo@unicampania.it

**Keywords:** energy harvester interfaces, double-stage converters, maximum power point tracking, rectifiers, voltage doublers, wireless sensor networks

## Abstract

An emerging class of electromagnetic energy harvesters, primarily designed to scavenge energy from human motion, sea waves, or sporadic mechanical vibrations, generates impulsive voltage waveforms rather than conventional sinusoidal ones. For such harvesters, usually employed for powering wireless sensor network nodes, this paper presents an in-depth investigation of double-stage AC/DC conversion interfaces aimed at evaluating and comparing their performance in energy extraction. Closed-form expressions are derived for widely adopted topologies consisting of full-bridge or voltage doubler rectifiers followed by DC/DC converters that exhibit either constant-voltage or constant-resistance behavior at their input terminals. The derived expressions provide valuable design insight, showing that no topology is universally preferable under all operating conditions; therefore, design guidelines are provided to support the selection and optimization of the most appropriate configuration. The analytical results show good agreement with measurements performed on interface prototypes assembled using off-the-shelf components, with a relative error below 4% at the maximum-energy points for all the tested configurations. The experimental validation confirms the accuracy of the developed models and their practical relevance for the analysis and design of interfaces for impulsive electromagnetic energy harvesters.

## 1. Introduction

Powering wireless sensor network nodes and Internet of Things devices using energy harvesters that scavenge energy from the surrounding environment has emerged as an effective and environmentally sustainable alternative to disposable batteries [1,2]. Among the various transduction mechanisms employed in energy harvesters (including piezoelectric, triboelectric, thermoelectric, pyroelectric, and electrostatic), electromagnetic transduction offers notable advantages in terms of harvested power, scalability, and structural simplicity [3,4]. In electromagnetic energy harvesters, ambient energy sources induce relative motion between a permanent magnet and a coil, generating electrical power via electromagnetic induction [5,6].

While traditional electromagnetic harvesters are typically designed to produce sinusoidal or near-sinusoidal output voltages with slowly varying amplitudes, increasing attention has recently focused on electromagnetic harvesters that generate bipolar impulsive voltage waveforms. These devices are particularly suitable for energy sources that cannot be efficiently captured by conventional sinusoidal harvesters. In such systems, the relative motion between the magnet and the coil is large enough to cause the magnetic coupling to vary from zero to its maximum value and then back to zero, leading to impulsive voltage profiles. These harvesters generally operate at very low frequencies and deliver lower power levels than their sinusoidal counterparts.

This class of bipolar impulsive harvesters includes devices exploiting coils mounted on vehicle wheels passing near fixed magnets [7]; cylindrical magnets sliding inside fixed coils for harvesting energy from human motion [8]; cylindrical magnets rolling inside fixed coils for scavenging energy from sea waves [9] or train-induced vibrations [10]; and spherical magnets rolling within curved tubes to harvest energy from human motion [11] or ocean waves [12]. Pendulum-based systems driven by human motion [13] or sea waves [14,15] also fall within this category. Moreover, harvesters originally designed for sinusoidal operation may exhibit impulsive behavior when subjected to irregular or nonperiodic excitations such as railway track vibrations or bridge oscillations [16,17].

Since these harvesters produce bipolar voltages, rectification is required to supply DC loads. Moreover, electrical load adaptation is desirable to maximize power transfer. The simplest electronic interface relies on a double-stage architecture consisting of an uncontrolled rectifier, implemented with passive diodes [18,19,20,21,22] or active diodes [8,23,24], followed by a DC/DC converter responsible for maximizing power extraction.

An alternative to this approach is the use of single-stage active AC/DC converters [25,26,27,28,29], such as dual-polarity boost/buck–boost converters or active full-bridge converters. Dual-polarity boost/buck–boost converters allow unidirectional power flow and can only emulate a resistive load at their input [30,31,32], whereas active full-bridge converters support bidirectional power transfer and can emulate complex impedances [33,34,35]. Although sinusoidal electromagnetic harvesters may benefit from such impedance emulation, the very low operating frequencies typical of impulsive harvesters render the imaginary part of their internal impedance negligible, making reactive impedance emulation unnecessary [8]. Moreover, single-stage active converters, although potentially more efficient, often require microcontrollers or custom integrated circuits to implement high-performance Maximum Power Point Tracking (MPPT) algorithms, thereby increasing power consumption or system cost. Consequently, double-stage solutions remain attractive for low-power, low-frequency impulsive electromagnetic energy harvesters, especially when cost constraints favor implementations based on off-the-shelf components [36].

Several studies have investigated the performance of rectifiers utilizing passive diodes, diode-connected MOSFETs, or active diode circuits [37,38]. The power extracted under steady-state sinusoidal excitation using full-bridge rectifiers with resistive-capacitive loads has been analyzed for both resonant [39] and non-resonant [40] harvesters, highlighting the influence of diode voltage drops. Alternative rectification topologies, such as voltage doublers and multipliers, have also been investigated [41]. Studies on optimal loading for doublers and multipliers have been conducted for piezoelectric [42] and RF energy harvesters [43,44] and later extended to N-stage multipliers [45].

However, these investigations, focused on sinusoidal excitations, do not directly extend to impulsive sources, as the behavior of nonlinear dynamic rectifiers (e.g., voltage doublers) strongly depends on the input waveform. Additionally, the influence of downstream converter control on rectifier performance has been neglected, limiting the availability of design guidelines for such interfaces.

To address this gap, this paper presents, for the first time to the best of the author’s knowledge, a comparative analysis of the energy extraction performance of double-stage conversion interfaces designed for impulsive electromagnetic harvesters. The examined topologies include a full-bridge rectifier and a voltage doubler, each followed by a DC/DC converter controlled to exhibit either a constant input voltage or a constant input resistance. Closed-form expressions relating the extracted energy to circuit parameters are derived, providing insight into the interface operation. The proposed analysis is experimentally validated through prototype measurements and offers a valuable tool for designing and optimizing electronic interfaces for low-frequency, low-power impulsive energy harvesters.

The rest of the paper is organized as follows: Section 2 introduces the model of an impulsive electromagnetic energy harvester and derives closed-form expressions for the extracted energy. Section 3 discusses design implications arising from the theoretical results. Section 4 presents the prototype implementation and experimental validation, followed by concluding remarks.

## 2. Materials and Methods

This section develops an analytical framework for impulsive electromagnetic energy harvesters and their power-conditioning interfaces. A general electromechanical model is first formulated for harvesters generating bipolar voltage pulses. The model is then used to derive closed-form expressions for the energy extracted by two-stage interfaces based on either a full-bridge rectifier or a voltage doubler, followed by a DC/DC converter operating as a constant-voltage or constant-resistance load. Finally, the scope and novelty of the proposed framework are discussed through a comparison with previously published analytical studies.

### 2.1. Model of an Impulsive Electromagnetic Harvester

When a permanent magnet and a coil come into proximity, such as when a cylindrical magnet approaches a coaxial coil, or when their axes become more closely aligned, as in the case of a spherical magnet rolling inside a coil, the magnetic flux linkage increases. Conversely, when they move apart or when their axes become orthogonal, the magnetic flux linkage decreases. Therefore, the passage of a magnet near a coil, or a change in their relative angular orientation, results in an increase followed by a decrease in the linked magnetic flux. This variation induces a voltage waveform consisting of two consecutive pulses of opposite polarity.

**Figure 1:**
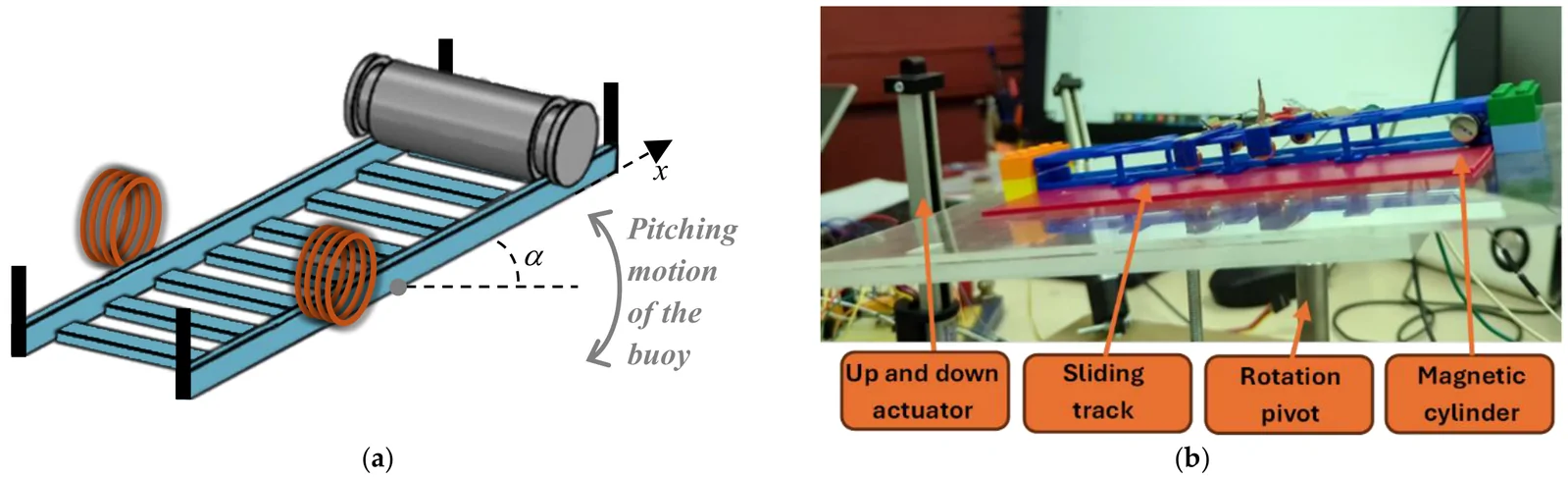
Sea-wave electromagnetic energy harvester consisting of a cylindrical permanent magnet rolling along a track and periodically passing near pairs of coils: (**a**) Schematic illustration. (**b**) Photo of a prototype [9].

Without loss of generality, consider an electromagnetic energy harvester designed to extract kinetic energy from the oscillatory motion of a buoy driven by sea waves. The system consists of a cylindrical magnet rolling along a track due to the wave-induced pitching motion of the buoy. As the magnet rolls, it periodically passes near pairs of coils symmetrically placed on both sides of the track. Figure 1 shows a schematic drawing and a photo of a prototype [9]. The dynamics of the magnet are governed by Newton’s second law:

(1) $$k_{I}\;m\;\ddot{x\;}=\;-m\;g\sin\alpha -\mu (x)\;\dot{x}$$

where x is the time-dependent position of the magnet’s center of mass along the track; $\dot{x}$ and $\ddot{x\;}$ are its velocity and acceleration, respectively; m is the magnet mass; g is the gravitational acceleration; α is the tilt angle of the track; and μ(x) is a position-dependent friction coefficient modeling the braking forces at the track’s ends. The coefficient $k_{I}=\;1+I_{cm}/\left(m\;R^{2}\right)$ accounts for the magnet’s rotational inertia, where $I_{cm}$ is the moment of inertia, and R is the radius of the magnet. Experimental results indicate that the Lorentz force due to the interaction between the coil current and the magnet’s magnetic field is negligible in this case [8,9,46].

The rolling motion of the magnet induces a time-varying magnetic flux through each pair of series-connected coils, generating an electromotive force $v_{s}$ and driving a current $i_{s}$ through the electrical load. The electromechanical behavior of the system is captured by

(2) $$\begin{array}{l} v_{s}=\theta (x)\;\dot{x}\\ v_{s}=R_{s}\;i_{s}+v_{H} \end{array}$$

Here, θ(x) denotes the electromechanical coupling coefficient, $R_{s}$ is the total internal resistance of the series-connected coils, and $v_{H}$ is the voltage across the harvester load. The current $i_{s}$ is defined as positive from the harvester toward the electrical load, and $v_{H}$ is defined according to the corresponding passive sign convention. Because sea-wave-induced motions occur at very low frequencies, the inductive reactance of the coils is much smaller than their winding resistance and can therefore be neglected [8,9,46].

**Figure 2:**
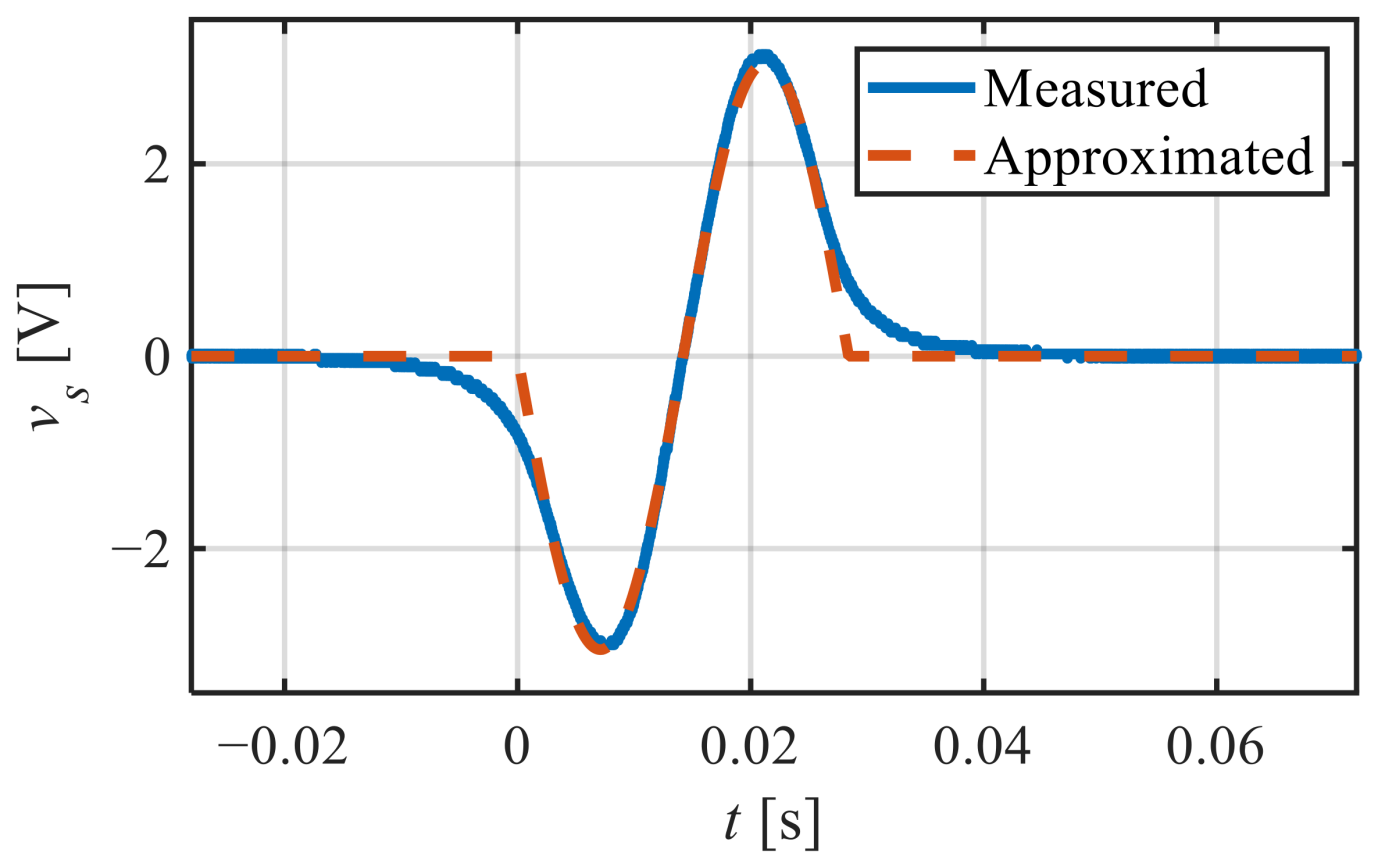
Measured open-circuit voltage generated by a series-connected coil pair of Figure 1, together with its sinusoidal approximation.

An example of a measured induced voltage waveform is shown in Figure 2. The waveform can be approximated as a single period of a sinusoid. Minor discrepancies between the measured and approximated signals near the tails are negligible, as their contribution to the total energy is minimal. Therefore, the induced voltage can be modeled as a single-period sinusoidal voltage source, $v_{s}=-V_{s}\sin\left(\omega \;t\right)$, in series with an internal resistance $R_{s}$, where $V_{s}$ is the peak voltage, ω=2π/T is the angular frequency, and T is the waveform duration. Although this model was originally presented in [46] for the sea-wave electromagnetic energy harvester introduced in [9], it is applicable to the broader class of impulsive electromagnetic energy harvesters, which generate bipolar voltage pulses that can be reasonably approximated by a single-period sinusoid.

### 2.2. Closed-Form Expressions

A theoretical analysis of the most common double-stage interfaces is presented here for electromagnetic energy harvesters generating bipolar impulsive voltages. The first stage consists of either a full-bridge rectifier or a voltage doubler, whereas the second stage is a DC/DC converter controlled to exhibit either a constant input voltage or a constant input resistance. The analysis is carried out using the harvester model introduced in the previous section and the constant voltage-drop model for the diodes.

It is shown that, while the full-bridge expressions also apply to steady-state continuous sinusoidal inputs, the voltage doubler expressions apply only to impulsive sources due to the different charge conditions of its capacitor.

#### 2.2.1. Full-Bridge Rectifier

**Figure 3:**
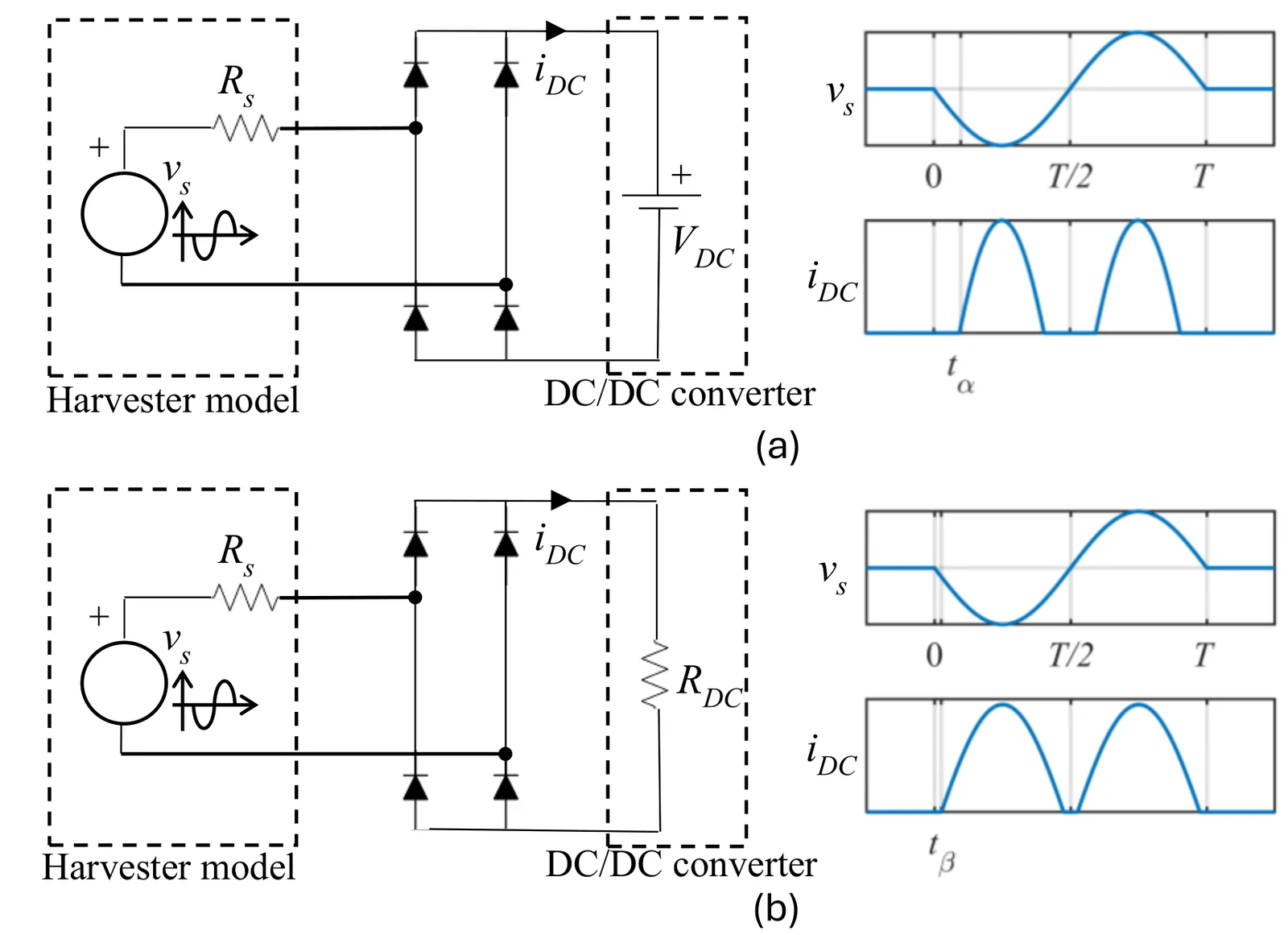
Schematic and operating waveforms of an impulsive electromagnetic energy harvester connected to a full-bridge rectifier followed by a DC/DC converter. The converter is controlled to exhibit either (**a**) a constant input voltage or (**b**) a constant input resistance.

Let us consider an impulsive electromagnetic energy harvester connected to a double-stage interface composed of a bridge rectifier followed by a DC/DC converter, as shown in Figure 3. According to the maximum power transfer theorem, the harvester delivers maximum power when the equivalent load resistance $R_{L}$ seen at its terminals matches the source resistance $R_{S}$. The energy transferred over the considered single period of the sinusoid is

(3) $$E_{ref}=\frac{{V_{s}}^{2}T}{8\;R_{s}}$$

This expression is used as a reference in the following derivations.

When the bridge rectifier is followed by a DC/DC converter that behaves as a constant voltage source $V_{DC}$, as shown in Figure 3a, the configuration is denoted *FBV.* The energy transferred from the rectifier to the converter over one period is

(4) $$E_{FBV}=V_{DC}\int_{0}^{T}i_{DC}dt$$

The current $i_{DC}$ is non-zero only when the absolute value of the source voltage exceeds $V_{DC}+2\;V_{D}$, where $V_{D}$ denotes the diode voltage drop. Thus, provided that ${V_{s}\geq V}_{DC}+2\;V_{D}$, diode conduction begins at

(5) $$t_{\alpha }=\frac{1}{\omega }\mathrm{asin}\left(\frac{V_{DC}+2V_{D}}{V_{s}}\right)$$

and the extracted energy can be written as

(6) $$E_{FBV}=2V_{DC}\int_{t_{\alpha }}^{T/2-t_{\alpha }}\frac{v_{s}-V_{DC}-2V_{D}}{R_{s}}dt$$

By solving the integral in (6) and normalizing the extracted energy with respect to the energy extracted by the optimal load, one obtains

(7) $$\bar{E_{FBV}}=\frac{16}{\pi }\bar{V_{DC}}\left[\sqrt{1-{\left(\bar{V_{DC}}+2\bar{V_{D}}\right)}^{2}}-\left(\bar{V_{DC}}+2\bar{V_{D}}\right)\left(\frac{\pi }{2}-\mathrm{asin}\left(\bar{V_{DC}}+2\bar{V_{D}}\right)\right)\right]$$

with $\bar{E_{FBV}}=E_{FBV}/E_{ref}$. The normalized DC voltage is $\bar{V_{DC}}=V_{DC}/V_{s}$ and the normalized diode voltage drop is $\bar{V_{D}}=V_{D}/V_{s}$.

It is worth noting that $\bar{E_{FBV}}$ does not represent the rectifier efficiency. Rather, it quantifies how closely the extracted energy approaches the maximum energy that could be delivered to an ideal matched load. In fact, even in the absence of rectifier losses (i.e., $V_{D}=0$), the value given by (7) remains below unity due to the inherent nonlinear behavior of the rectifier.

Next, consider the configuration denoted *FBR*, where the full-bridge rectifier is connected to a DC/DC converter that exhibits a constant input resistance $R_{DC}$, as shown in Figure 3b. The energy transferred from the full-bridge rectifier to its load over one period is

(8) $$E_{FBR}=R_{DC}\int_{0}^{T}{i_{DC}}^{2}dt$$

Here, the current $i_{DC}$ remains zero until the absolute value of the source voltage exceeds two diode voltage drops. Thus, provided that $V_{s}\geq 2\;V_{D}$, the conduction begins at the instant

(9) $$t_{\beta }=\frac{1}{\omega }\mathrm{asin}\left(2\frac{V_{D}}{V_{s}}\right)$$

Thus, the extracted energy in (8) can be written as

(10) $$E_{FBR}=\frac{2R_{DC}}{{\left(R_{s}+R_{DC}\right)}^{2}}\int_{t_{\beta }}^{T/2-t_{\beta }}{\left[V_{s}\sin\left(\omega t\right)-2V_{D}\right]}^{2}dt$$

After solving the integral in (10), the normalized energy $\bar{E_{FBR}}=E_{FBR}/E_{ref}$ can be calculated as

(11) $$\bar{E_{FBR}}=\frac{4\bar{R_{DC}}}{{\left(1+\bar{R_{DC}}\right)}^{2}}\left[\left(1+8{\bar{V_{D}}}^{2}\right)\left(1-\frac{2}{\pi }\mathrm{asin}\left(2\bar{V_{D}}\right)\right)-\frac{12}{\pi }\bar{V_{D}}\sqrt{1-4{\bar{V_{D}}}^{2}}\right]$$

with $\bar{R_{DC}}=R_{DC}/R_{s}$.

#### 2.2.2. Voltage Doubler

**Figure 4:**
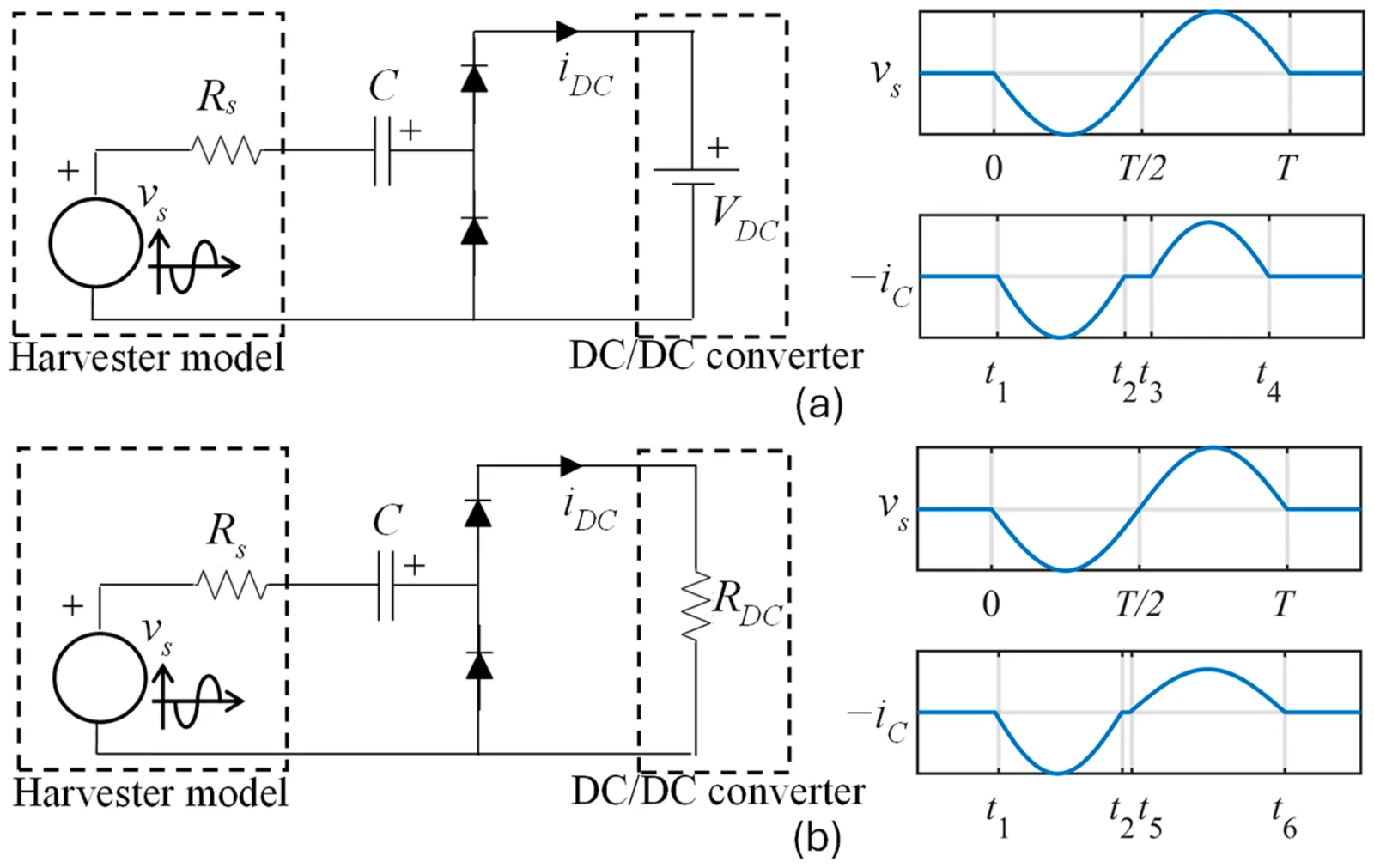
Schematic and operating waveforms of an impulsive electromagnetic energy harvester connected to a voltage doubler followed by a DC/DC converter. The converter is controlled to exhibit either (**a**) a constant input voltage or (**b**) a constant input resistance.

Let us consider now an impulsive electromagnetic energy harvester connected to a voltage doubler followed by a DC/DC converter. The converter is controlled to exhibit either a constant input voltage (Figure 4a) or a constant input resistance (Figure 4b). In both cases, the operation of the voltage doubler consists of two distinct phases.

During the first phase, when the source voltage is negative, the lower diode becomes forward-biased, allowing the capacitor C to charge. In this interval, the upper diode is reverse-biased, and the downstream converter receives no power. Charging continues until the current flowing into the capacitor becomes zero. During the second phase, the current in the capacitor reverses direction, forward-biasing the upper diode. The downstream converter is then supplied by the source and the capacitor. This phase ends when the capacitor current again reaches zero.

In the first phase, the circuit is governed by the following differential equation:

(12) $$R_{s}C\frac{dv_{c}}{dt}+v_{c}=-v_{s}-V_{D}$$

with the initial condition that the capacitor charging begins when the absolute value of the source voltage exceeds the sum of the lower diode voltage drop and the initial capacitor voltage $V_{c0}$, i.e.,

(13) $$v_{c}\left(t_{1}\right)=V_{c0}\;t_{1}=\frac{1}{\omega }\mathrm{asin}\frac{V_{c0}+V_{D}}{V_{s}}$$

provided that V_s ≥ V_c0 + V_D. Solving (12) and (13) yields

(14) $$v_{c}\left(t\right)=\left(V_{c0}+V_{D}-\frac{V_{s}}{\sqrt{1+\delta^{2}}}\sin\left(\omega t_{1}-\theta_{s}\right)\right)e^{-\frac{t-t_{1}}{CR_{s}}}+\frac{V_{s}}{\sqrt{1+\delta^{2}}}\sin\left(\omega t-\theta_{s}\right)-V_{D}$$

where $\delta =2\pi \;C\;R_{s}/T$ and $\theta_{s}=\mathrm{atan}\delta$.

According to (14), the capacitor voltage increases until it reaches its maximum value, at which point diode conduction stops. The voltage maximum is obtained by differentiating (14), setting the derivative to zero, and solving for the corresponding time. Since the peak is expected to occur near the maximum of the sinusoidal term $\sin\left(\omega t-\theta_{s}\right)$, the exponential term in (14) is approximated by a first-order Taylor expansion around the time at which the sinusoidal term reaches its maximum, i.e.,

(15) $$t_{x}=\frac{1}{\omega }\left(\frac{\pi }{2}+\theta_{s}\right)$$

Thus, the exponential term in (14) can be written as

(16) $$e^{-\frac{t-t_{1}}{CR_{s}}}=e^{-\frac{1}{\delta }\left(\frac{\pi }{2}+\theta_{s}\right)+\frac{\omega t_{1}}{\delta }}-\frac{e^{-\frac{1}{\delta }\left(\frac{\pi }{2}+\theta_{s}\right)+\frac{\omega t_{1}}{\delta }}}{CR_{s}}\left(t-t_{x}\right)$$

By substituting (16) into (14), differentiating the resulting expression with respect to time, setting the derivative to zero, and solving the equation, the time at which the capacitor voltage reaches its maximum can be determined, namely,

(17) $$t_{2}=\frac{1}{\omega }\left(\frac{\pi }{2}+\theta_{s}-\varepsilon \right)$$

with

(18) $$\varepsilon =\mathrm{asin}\left[\left(\sin\left(\mathrm{atan}\delta -\mathrm{asin}\left(\bar{V_{c0}}+\bar{V_{D}}\right)\right)+\right.\left.\left(\bar{V_{c0}}+\bar{V_{D}}\right)\sqrt{1+\delta^{2}}\right)\cdot \frac{e^{-\frac{1}{\delta }\left(\frac{\pi }{2}+\mathrm{atan}\delta -\mathrm{asin}\left(\bar{V_{c0}}+\bar{V_{D}}\right)\right)}}{\delta }\right]$$

and $\bar{V_{c0}}=V_{c0}/V_{s}$. The maximum value of the capacitor voltage (14) is obtained at $t_{2}$ and is equal to

(19) $$\bar{V_{cM}}=\left(\bar{V_{c0}}+\bar{V_{D}}+\frac{\sin\left(\mathrm{atan}\delta -\mathrm{asin}\left(\bar{V_{c0}}+\bar{V_{D}}\right)\right)}{\sqrt{1+\delta^{2}}}\right)e^{-\frac{1}{\delta }\left(\frac{\pi }{2}+\mathrm{atan}\delta -\mathrm{asin}\left(\bar{V_{c0}}+\bar{V_{D}}\right)-\varepsilon \right)}\;+\frac{\cos\varepsilon }{\sqrt{1+\delta^{2}}}-\bar{V_{D}}$$

where $\bar{V_{cM}}=\max\left(v_{c}(t)\right)/V_{s}$.

In the second phase of the voltage doubler operation, the upper diode is biased forward, and its current depends on the behavior of the DC/DC converter. Let us first consider the case of a voltage doubler connected to a DC/DC converter controlled to exhibit a constant input voltage. In this configuration, denoted *VDV*, the converter can be modeled as a voltage source, as shown in Figure 4a. During this phase, the circuit is described by the following differential equation:

(20) $$R_{s}C\frac{dv_{c}}{dt}+v_{c}=-v_{s}+V_{D}+V_{DC}$$

with the initial condition that the current $i_{DC}$, which is equal and opposite to the capacitor current $i_{C}$, i.e., $i_{DC}=-i_{C}$, remains zero until the sum of the source voltage and the capacitor voltage exceeds $V_{DC}+V_{D}$. The instant $t_{3}$, at which $dv_{c}/dt=0$, is given by

(21) $$\frac{dv_{c}}{dt}\left(t_{3}\right)=0\;\;\;\;\;\;\;\;t_{3}=\frac{1}{\omega }\left(\pi -\mathrm{asin}\frac{V_{cM}-V_{D}-V_{DC}}{V_{s}}\right)$$

provided that $V_{s}+V_{cM}\geq V_{DC}+V_{D}$. This condition ensures that the argument of the asin function in (21) is greater than or equal to −1. Furthermore, since the capacitor voltage cannot exceed $V_{s}-V_{D}$ during the first phase, $V_{cM}\leq V_{s}-V_{D}$, and therefore the argument of the asin function in (21) is less than 1. The solution of (20) yields a closed-form expression for $i_{DC}$, i.e.,

(22) $$i_{DC}(t)=-\frac{V_{s}\;\omega \;C}{\sqrt{1+\delta^{2}}}\left[\cos\left(\omega \;t-\theta_{s}\right)-\cos\left(\omega \;t_{3}-\theta_{s}\right)e^{-\frac{t-t_{3}}{C\;R_{s}}}\right]$$

The $i_{DC}$ current in (22) delivers power to the DC/DC converter until it reaches zero. By neglecting the exponential decay in (22), the conduction end time can be written as

(23) $$t_{4}=\frac{1}{\omega }\left(2\pi -\omega \;t_{3}+2\;\theta_{s}\right)$$

and the energy delivered to the converter can be calculated as

(24) $$E_{VDV}=V_{DC}\int_{t_{3}}^{t_{4}}i_{DC}dt$$

By solving (24) using the expressions in (21)–(23), the energy extracted by a voltage doubler followed by a DC/DC converter exhibiting a constant input voltage can be calculated as

(25) $$\bar{E_{VDV}}=\frac{8}{\pi }\frac{\delta \;\;\bar{V_{DC}}}{\sqrt{1+\delta^{2}}}\left[\sin\left(\mathrm{asin}\left(\bar{V_{cM}}-\bar{V_{D}}-\bar{V_{DC}}\right)+\mathrm{atan}\delta \right)-\left(1-e^{-\frac{\Delta_{34}}{\delta }}\right)\frac{\delta }{2}\cos\left(\mathrm{asin}\left(\bar{V_{cM}}-\bar{V_{D}}-\bar{V_{DC}}\right)+\mathrm{atan}\delta \right)\right]$$

with $\Delta_{34}=2\mathrm{asin}\left(\bar{V_{cM}}-\bar{V_{D}}-\bar{V_{DC}}\right)+2\mathrm{atan}\delta$ and $\bar{E_{VDV}}=E_{VDV}/E_{ref}$.

Let us now analyze the second phase of the voltage doubler operation for the case where the voltage doubler is connected to a DC/DC converter controlled to exhibit a desired input resistance (*VDR*). In this scenario, the converter can be modeled as shown in Figure 4b. Accordingly, the circuit behavior is described by the following differential equation:

(26) $$\left(R_{s}+R_{DC}\right)\;C\frac{dv_{c}}{dt}+v_{c}=-v_{s}+V_{D}$$

with the initial condition that the current $i_{DC}$, which is equal and opposite to the capacitor current $i_{C}$, i.e., $i_{DC}=-i_{C}$, remains zero until the sum of the source voltage and the capacitor voltage exceeds $V_{D}$. The instant $t_{5}$, at which $dv_{c}/dt=0$, is given by

(27) $$\frac{dv_{c}}{dt}\left(t_{5}\right)=0\;t_{5}=\frac{1}{\omega }\left(\pi -\mathrm{asin}\frac{V_{cM}-V_{D}}{V_{s}}\right)$$

provided that $V_{s}+V_{cM}\geq V_{D}$. This condition ensures that the argument of the asin function in (27) is greater than or equal to −1. Furthermore, since the capacitor voltage cannot exceed $V_{s}-V_{D}$ during the first phase, $V_{cM}\leq V_{s}-V_{D}$, and therefore the argument of the asin function in (27) is less than 1. By solving (26) and (27), the expression of $i_{DC}$ is obtained:

(28) $$i_{DC}(t)=-\frac{V_{s}\omega C}{\sqrt{1+\delta_{D}^{2}}}\left[\cos\left(\omega t-\theta_{D}\right)-\cos\left(\omega t_{5}-\theta_{D}\right)e^{-\frac{t-t_{5}}{C(R_{s}+R_{DC})}}\right]$$

where $\delta_{D}=\delta \;(1+\bar{R_{DC}})$, $\bar{R_{DC}}=R_{DC}/R_{s}$, $\theta_{D}=\mathrm{atan}\delta_{D}$. The current $i_{DC}$ flows through the resistor $R_{DC}$ until it drops to zero at time $t_{6}$, which can be calculated by neglecting the exponential decay in (28) as follows:

(29) $$t_{6}=\frac{1}{\omega }\left(2\pi -\omega t_{5}+2\theta_{D}\right)$$

To predict the extracted energy,

(30) $$E_{VDR}=R_{DC}\int_{t_{5}}^{t_{6}}{i_{DC}}^{2}dt$$

the exponential term in (28) is approximated using a first-order Taylor expansion around $t=t_{5}$, i.e.,

(31) $$e^{-\frac{t-t_{5}}{C(R_{s}+R_{DC})}}=1-\frac{1}{C(R_{s}+R_{DC})}\left(t-t_{5}\right)$$

Accordingly, the extracted energy can be expressed as

(32) $$E_{VDR}=R_{DC}\frac{{\left(V_{s}\omega C\right)}^{2}}{1+\delta_{D}^{2}}\int_{t_{5}}^{t_{6}}{\left[\cos\left(\omega t-\theta_{D}\right)-\cos\left(\omega t_{5}-\theta_{D}\right)\left(1-\frac{1}{C(R_{s}+R_{DC})}\left(t-t_{5}\right)\right)\right]}^{2}dt$$

Consequently, the energy extracted by a voltage doubler followed by a DC/DC converter exhibiting a constant input resistance is given by (33).

(33) $$\bar{E_{VDR}}=\frac{2\delta^{2}\bar{R_{DC}}{\left(3\pi \right)}^{-1}}{1+\delta^{2}{\left(1+\bar{R_{DC}}\right)}^{2}}\left[4\alpha \left(3-3\frac{\alpha }{\delta_{d}}+2\frac{\alpha^{2}}{{\delta_{d}}^{2}}\right)+2\alpha \left(3-6\frac{\alpha }{\delta_{d}}+4\frac{\alpha^{2}}{{\delta_{d}}^{2}}\right)\cos\left(2\alpha \right)-3\left(3-4\frac{\alpha }{\delta_{d}}\right)\sin\left(2\alpha \right)\right]\;\;\alpha =\mathrm{atan}\left(\delta \left(1+\bar{R_{DC}}\right)\right)+\mathrm{asin}\left(\bar{V_{cM}}-\bar{V_{D}}\right)$$

with $\bar{E_{VDR}}=E_{VDR}/E_{ref}$.

It should be noted that, to derive expressions (25) and (33), Taylor series expansions were truncated at the zeroth or first order in the derivations of (16), (23), (29), and (31). The accuracy of these approximations improves as the corresponding circuit time constants become large compared with the waveform duration T. In (16) and (23), the relevant time constant is $CR_{s}$, whereas in (29) and (31), it is $C(R_{s}+R_{DC})$. Since $C\left(R_{s}+R_{DC}\right)>CR_{s}$, the condition $CR_{s}/T\geq 1$ provides a conservative criterion for all the Taylor approximations considered here. The comparison presented in Section 4 between the measured extracted energies and the values predicted by the complete analytical expressions shows that, for the tested configurations with $CR_{s}/T\geq 1$, the relative error is lower than 4% in all cases. This result provides a quantitative assessment of the overall accuracy of the analytical expressions under the investigated operating conditions. As $CR_{s}/T$ increases, the Taylor approximations are expected to become more accurate, thereby reducing their contribution to the overall approximation error.

It is worth noting that, to the best of the author’s knowledge, this is the first time that the energy extracted by a voltage doubler, whether followed by a constant voltage source (25) or by a resistive load (33), is expressed in a closed form as a function of the circuit parameters.

According to (25) and (33), the extracted energy depends on the maximum capacitor voltage $V_{cM}$, which in turn depends on the initial capacitor voltage $V_{c0}$. In the case of steady-state trains of isolated bipolar pulses, $V_{c0}$ can be estimated as follows:

For a voltage doubler followed by a resistive load, the capacitor discharges its stored charge through $R_{DC}$ between consecutive pulses. For sufficiently long intervals between consecutive pulses compared with the discharge time constant, the capacitor can be assumed to fully discharge. Therefore, the initial capacitor voltage at the beginning of each pulse can be assumed to be zero, i.e., $V_{c0}=0$. For shorter pulse repetition intervals, the capacitor may retain a non-zero residual voltage between consecutive pulses. In this case, $V_{c0}$ depends on the pulse repetition interval.

In contrast, for a voltage doubler followed by a voltage source, the capacitor does not fully discharge because the upper diode turns off when the voltage at its anode falls below $V_{DC}$. As a result, a residual charge remains on the capacitor until the next pulse. By averaging over the pulse period T the equations of the two loops of the circuit in Figure 4a, and assuming equal average voltage drops across the two diodes, the average voltage across the capacitor is found to be $V_{DC}/2$. A first estimate of the maximum value of the capacitor voltage, denoted as $V_{cM}^{\prime }$, can therefore be obtained by applying (19) with $V_{c0}=V_{DC}/2$, i.e., by using the mean value as the initial condition. A refined estimate can be obtained by applying (19) with $V_{c0}=V_{DC}/2-(V_{cM}^{\prime }-V_{DC}/2)/2$, which corresponds to shifting the initial voltage by half of its deviation from the mean value.

### 2.3. Comparison with Previous Analytical Studies

Table 1 summarizes the main differences between the proposed analytical framework and previously published studies on the analysis and design of double-stage interfaces for energy harvesters. To the best of the author’s knowledge, this is the first analytical study to investigate the performance of a voltage doubler when driven by an impulsive electromagnetic energy harvester. Previous analyses of both full-bridge rectifiers and voltage doublers have been limited to continuous sinusoidal sources, whereas this work extends the analysis to impulsive electromagnetic sources. The impulsive nature of the source significantly increases the analytical complexity because the constant-capacitor-voltage assumption commonly adopted for voltage doublers under sinusoidal steady-state operation is no longer valid [44]. Furthermore, to the best of the author’s knowledge, this is the first analytical study to compare constant-input-voltage and constant-input-resistance DC/DC converters when combined with both rectifier topologies.

## 3. Design Guidelines

In the previous section, closed-form expressions were derived for the energy extracted by the four electronic interfaces connected to impulsive electromagnetic energy harvesters. To gain insight into the behavior of these circuits, it is useful to observe that, according to (7), (11), (25), and (33), the extracted energy depends on only two quantities in the case of the bridge rectifier and on three quantities in the case of the voltage doubler. Specifically, the functional dependencies are

(34) $$\begin{array}{l} \bar{E_{FBV}}=\bar{E_{FBV}}\left(V_{D}/V_{s},V_{DC}/V_{s}\right)\\ \bar{E_{FBR}}=\bar{E_{FBR}}\left(V_{D}/V_{s},R_{DC}/R_{s}\right)\\ \bar{E_{VDV}}=\bar{E_{VDV}}\left(V_{D}/V_{s},V_{DC}/V_{s},CR_{s}/T\right)\\ \bar{E_{VDR}}=\bar{E_{VDR}}\left(V_{D}/V_{s},R_{DC}/R_{s},CR_{s}/T\right) \end{array}$$

where the dependence on $CR_{s}/T$ is through $\delta =2\pi CR_{s}/T$. Note that $V_{s}$ and T depend on the ambient source, $R_{s}$ depends on the harvester coils, while $V_{D}$ depends on the rectifier implementation.

**Figure 5:**
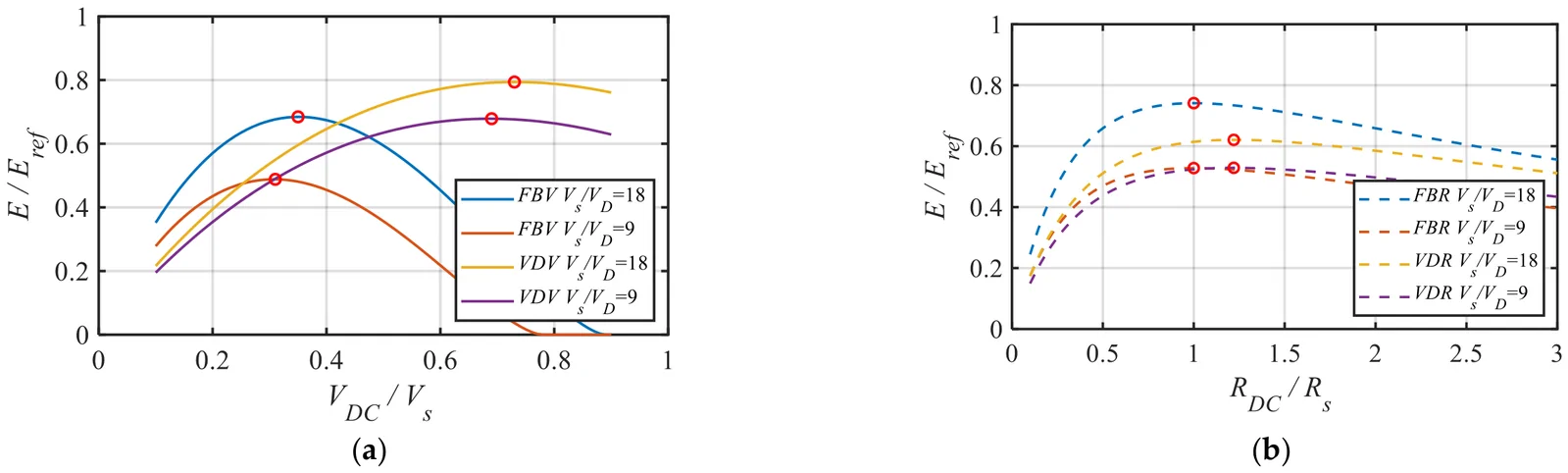
Analytical predictions: (**a**) extracted energy for the *FBV* and *VDV* interfaces as a function of the input voltage set by the DC/DC converter; (**b**) extracted energy for the *FBR* and *VDR* interfaces as a function of the input resistance set by the DC/DC converter. In all plots, red circles denote the maximum points, and $CR_{s}/T$ is set to 1.

Figure 5 shows the trends of the normalized extracted energy for the four considered topologies as a function of the quantity regulated by the DC/DC converter, namely, $V_{DC}$ or $R_{DC}$. Figure 5a illustrates the energy extracted by a full-bridge or a voltage doubler followed by a DC/DC converter with a constant input voltage, plotted as a function of $V_{DC}$ for two values of $V_{s}/V_{D}$. The results indicate that the optimal ratio $V_{DC}/V_{s}$, denoted by red circles, is nearly constant as $V_{s}/V_{D}$ varies, for both *FBV* and *VDV*. This implies that the optimal $V_{DC}$ set by the DC/DC converter should vary according to the pulse peak value $V_{s}$. Consequently, an MPPT controller that dynamically adjusts $V_{DC}$ is required to ensure maximum energy extraction.

However, Figure 5a reveals that the optimal fraction $V_{DC}/V_{s}$ is not exactly constant as $V_{s}/V_{D}$ varies. Therefore, the MPPT controller cannot keep this ratio constant but should adapt it to the characteristics of both the source and the rectifier to accurately track the true maximum power point. This observation is crucial for MPPT methods based on the Fractional Open-Circuit Voltage technique, as it highlights that these methods cannot reach the actual maximum power point under all operating conditions if the voltage fraction is kept constant, as usual.

Figure 5b reports the extracted energy for topologies employing a second stage with a constant resistive input. Unlike the behavior observed in Figure 5a, the optimal value of $R_{DC}$, corresponding to the red circles, remains constant across different values of $V_{s}/V_{D}$. This indicates that the optimal resistance is independent of the source and depends solely on the energy harvester characteristics, through $R_{s}$. It can therefore be determined during an initial calibration and kept constant during normal operation. Although harvester parameters may slowly vary because of temperature, aging, or other long-term effects, these variations occur over time scales much longer than the electrical dynamics, while the extracted-energy curves exhibit limited sensitivity around their maximum.

Consequently, for interfaces with constant-input-resistance converters, unlike those with constant-input-voltage converters, continuous MPPT operation can be omitted in low-power applications. This offers the benefits of reduced power consumption associated with the MPPT circuitry, particularly when the MPPT algorithm requires a microcontroller, and reduced implementation cost when dedicated MPPT hardware is needed. Moreover, for impulsive sources characterized by pulses separated by zero-input intervals, conventional MPPT techniques may be unreliable because voltage or power measurements performed during pulse-free intervals are not representative of the source behavior, potentially leading to incorrect operating-point updates. Should long-term parameter drifts eventually shift the optimal operating point, an occasional recalibration procedure can be performed. Since these variations occur over very long time scales, the calibration routine can remain inactive during normal operation and be activated only at infrequent intervals, selected according to the expected rate of parameter variations. The resulting average power consumption is therefore negligible compared with that of continuous MPPT algorithms. Therefore, the trends in Figure 5 highlight the critical role of the second stage in achieving and maintaining maximum energy extraction; in this regard, the *FBR* and *VDR* topologies provide an alternative that avoids continuous MPPT operation.

Let us now observe that, while $V_{DC}$ and $R_{DC}$ are set by the DC/DC converter, either dynamically under MPPT or statically when preset, the other quantities affecting the extracted energy, namely, the diode voltage drop $V_{D}$, the impulse peak $V_{s}$, and $CR_{s}/T$ are inherent to the circuit and source. Thus, it is useful to analyze the maximum energy extractable by each topology when $V_{DC}$ or $R_{DC}$ are optimally set. The maximum extractable energy under MPPT operation can be defined as

(35) $$\begin{array}{l} \bar{E_{FBV\text{\_}max}}\left(V_{D}/V_{s}\right)=\underset{\;V_{\mathrm{DC}}/V_{s}}{\max}\bar{E_{FBV}}\\ \bar{E_{FBR\text{\_}max}}\left(V_{D}/V_{s}\right)=\underset{R_{\mathrm{DC}}/R_{s}}{\max}\bar{E_{FBR}}\\ \bar{E_{VDV\text{\_}max}}\left(V_{D}/V_{s},\;CR_{s}/T\right)=\underset{V_{\mathrm{DC}}/V_{s}}{\max}\bar{E_{VDV}}\\ \bar{E_{VDR\text{\_}max}}\left(V_{D}/V_{s},\;CR_{s}/T\right)=\underset{R_{\mathrm{DC}}/R_{s}}{\max}\bar{E_{VDR}} \end{array}$$

**Figure 6:**
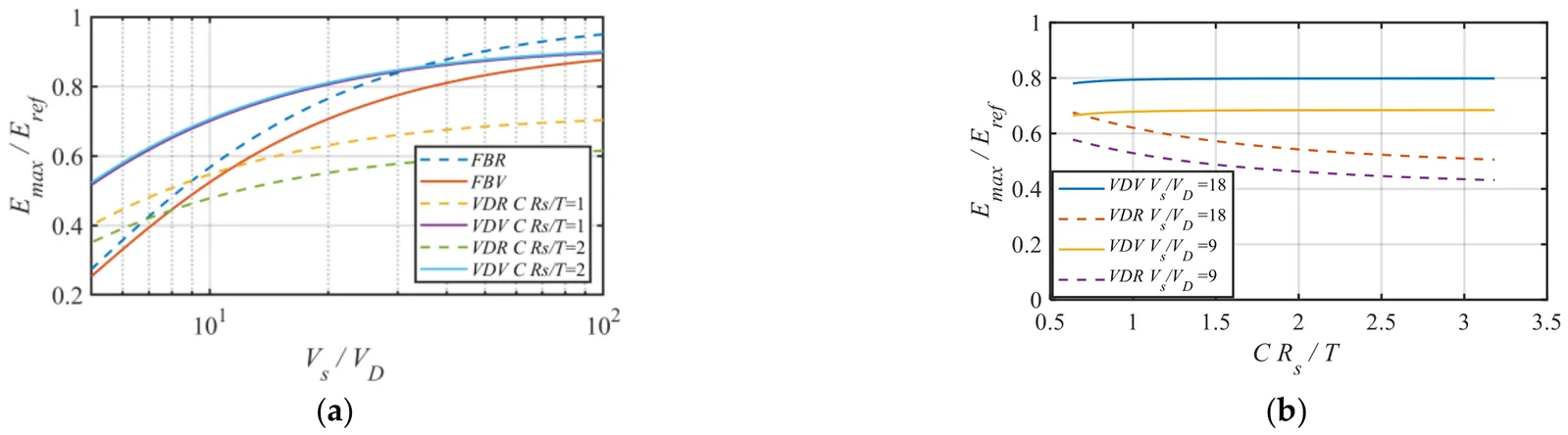
Analytical predictions: (**a**) maximum energy extractable by the four considered interfaces (*FBV*, *FBR*, *VDV*, and *VDR*) as a function of $V_{s}/V_{D}$; (**b**) maximum energy extractable by the voltage doubler-based interfaces as a function of $CR_{s}/T$.

Figure 6a shows these maximum energies as functions of $V_{s}/V_{D}$ for two values of $CR_{s}/T$. When the source voltage $V_{s}$ is high and the diode voltage drop $V_{D}$ is low, the full-bridge rectifier followed by a resistance, *FBR*, extracts the highest energy. Conversely, for $V_{s}/V_{D}$ below approximately 30, the *VDV* topology becomes superior. However, as discussed earlier, resistive-input converters eliminate the need for a continuous MPPT to compensate for variations in $V_{s}$. Thus, focusing on resistive-input converters, the voltage doubler topology *VDR* outperforms the full-bridge topology *FBR* up to $V_{s}/V_{D}\approx 10$. Therefore, Figure 6a enables the identification of the most suitable topology by considering the characteristics of both the harvester and the source, as well as the rectifier implementation using either passive or active diodes.

Figure 6b examines the influence of $CR_{s}/T$ on the maximum extracted energy for *VDV* and *VDR*. The performance of the interface with a constant-input-voltage converter remains nearly constant as $CR_{s}/T$ increases, whereas that with a constant-input-resistance converter shows a slight decrease. Thus, for *VDR*, smaller values of $CR_{s}/T$ are preferable.

The presented analysis can be directly used as a design guideline for interface selection. First, the harvester parameters $R_{s}$, $V_{s}$, and T, together with the rectifier voltage drop $V_{D}$, should be estimated. The ratios $V_{s}/V_{D}$ and $CR_{s}/T$ are then evaluated, and Figure 6a is used to identify the topology that provides the highest extractable energy. Nevertheless, the choice should also account for implementation aspects. While constant-input-voltage converters may achieve higher energy extraction in some operating regions, they require MPPT control to continuously adapt the operating point to source variations. Conversely, constant-input-resistance converters can maintain a fixed operating condition that is independent of variations in $V_{s}$ and can often eliminate the need for continuous MPPT, resulting in lower control complexity, reduced power consumption, and avoiding potential MPPT issues when processing impulsive waveforms. Once the topology has been selected, Figure 5 provides the optimum operating condition for the second stage. For constant-input-resistance converters, the optimum value of $R_{DC}$ can be determined offline and kept fixed during operation, whereas constant-input-voltage converters require MPPT to continuously adjust $V_{DC}$. Finally, Figure 6b can be used, when applicable, to optimize the capacitance value of voltage-doubler interfaces. Therefore, the proposed analytical framework provides not only performance prediction but also a systematic methodology for practical interface selection and design.

## 4. Experimental Results

**Figure 7:**
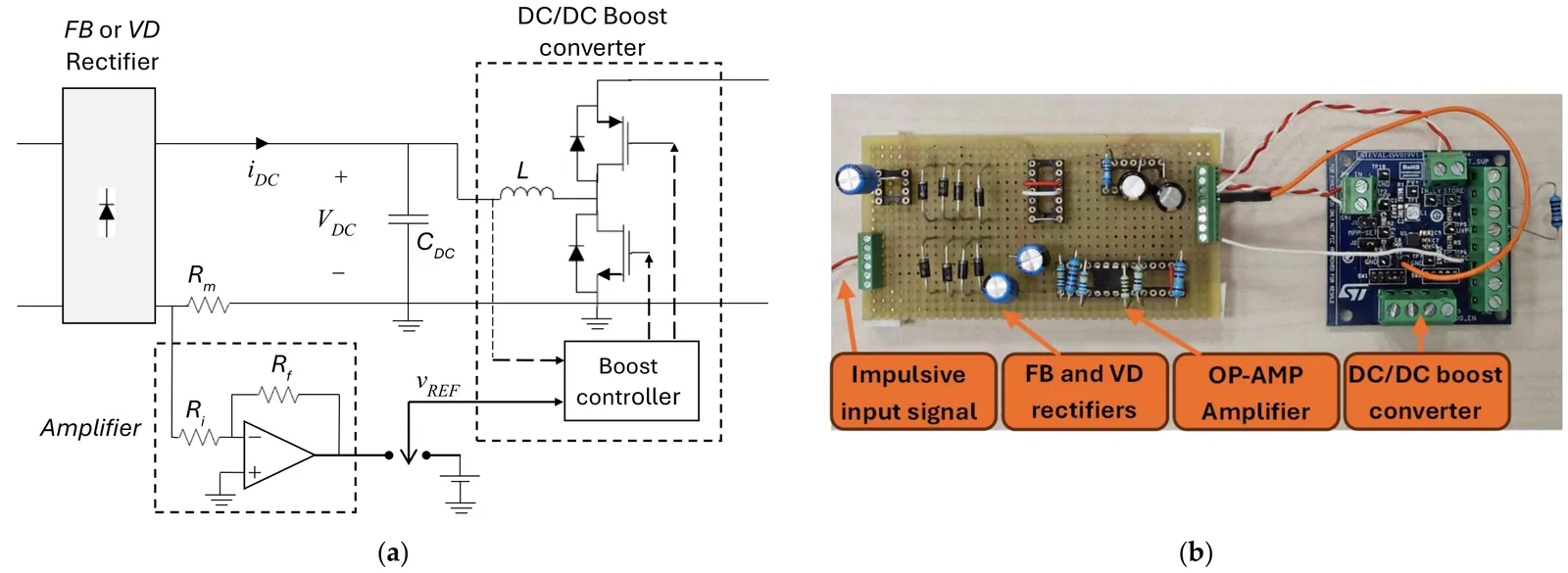
Implemented double-stage interfaces consisting of a rectifier and a DC/DC converter: (**a**) Schematic diagram. (**b**) Photo of the prototype.

To validate the theoretical developments, the four electronic interfaces shown in Figure 3 and Figure 4 were implemented and experimentally tested. The full-bridge and voltage doubler rectifiers were assembled using 1N5817 Schottky diodes (Vishay, Malvern, PA, USA), whereas the DC/DC converter was implemented using the SPV1050 integrated synchronous boost converter by STMicroelectronics (Geneva, Switzerland). The SPV1050 controller accepts an input reference voltage, $v_{REF}$, which is used to regulate the converter’s input voltage. For the configurations employing a constant input voltage converter, a fixed reference voltage was applied directly to the controller input. Conversely, for the configurations employing a constant input resistance converter, the reference voltage $v_{REF}$ was generated by sensing the DC current $i_{DC}$ through a measurement resistance $R_{m}$ and amplifying the corresponding voltage. According to the schematic of the implemented double-stage interfaces shown in Figure 7a, the amplifier output provides the reference voltage

(36) $$v_{REF}=\frac{R_{f}R_{m}}{R_{i}}\;i_{DC}$$

Since the converter ensures $V_{DC}=v_{REF}$, the ratio $V_{DC}/i_{DC}$ equals the coefficient $R_{f}R_{m}/R_{i}$. Hence, this coefficient defines the converter input resistance: $R_{DC}=R_{f}R_{m}/R_{i}$. The value of $R_{i}$ is adjusted to obtain the desired input resistance $R_{DC}$. The component values used in the experimental setup are: $R_{s}=350\;\Omega$, C=100 μF, $C_{DC}=9.4\;\mu F$, $R_{m}=10\;\Omega$, $R_{f}=5.56\;M\Omega$ and the boost-converter inductance L=22 μH. The harvester windings have an internal inductance of approximately $L_{s}=20\;mH$, for which $\omega L_{s}/R_{s}$ is 1.8% and 0.9% for T=20 ms and T=40 ms, respectively, indicating that the inductive contribution is negligible under the considered operating conditions. A photo of the implemented interfaces is shown in Figure 7b.

**Figure 8:**
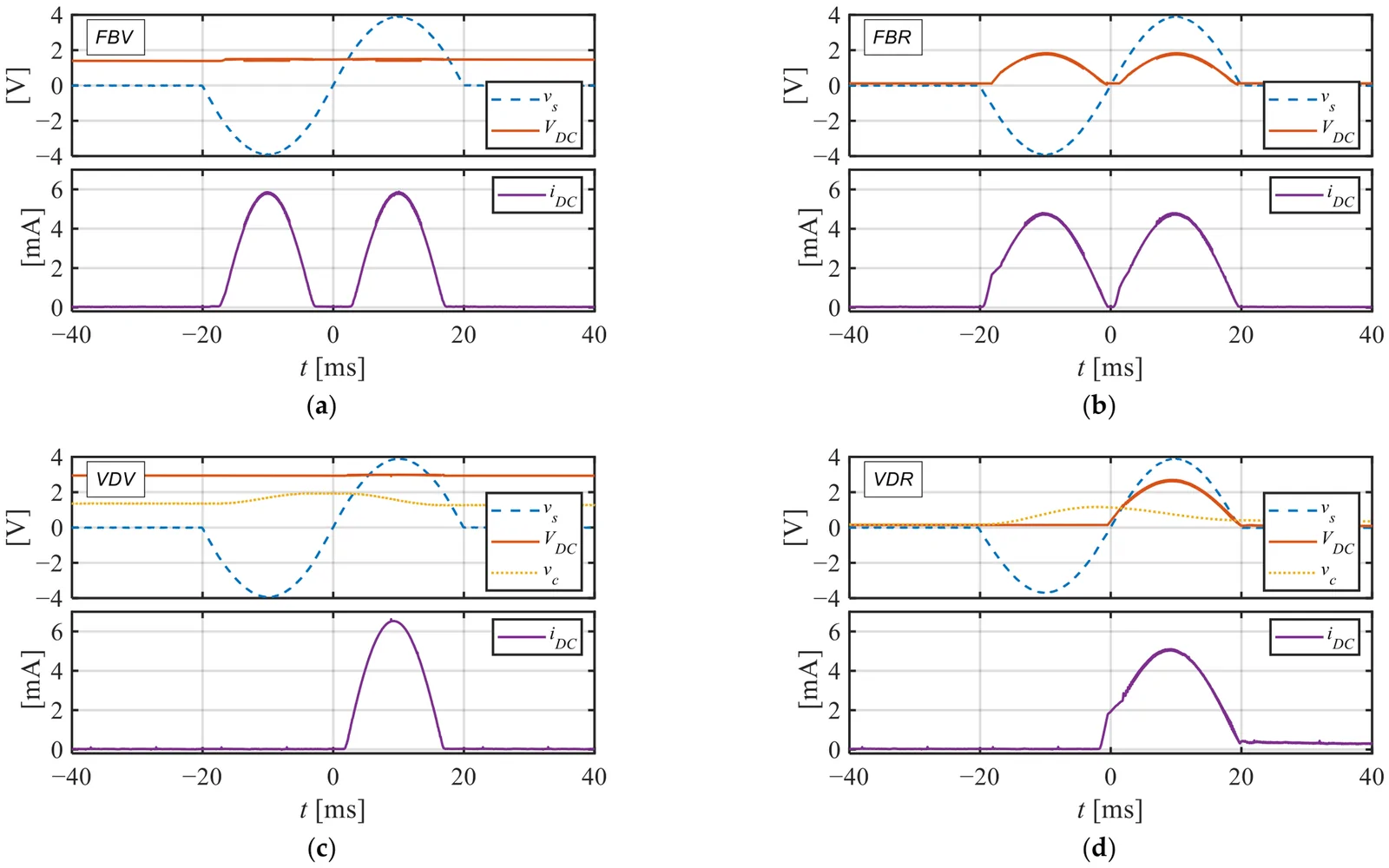
Measured time waveforms of source voltage $v_{s}$, DC voltage $V_{DC}$, voltage doubler capacitor voltage $v_{c}$ and DC current $i_{DC}$: (**a**) Full-bridge rectifier with a converter exhibiting a constant input voltage ($v_{REF}=1.5\;V$). (**b**) Full-bridge rectifier with a converter exhibiting a constant input resistance ($R_{DC}=390\;\Omega$). (**c**) Voltage doubler with a converter exhibiting a constant input voltage ($v_{REF}=3\;V$). (**d**) Voltage doubler with a converter exhibiting a constant input resistance ($R_{DC}=560\;\Omega$).

Figure 8 reports the measured waveforms of the source voltage $v_{s}$, the DC voltage $V_{DC}$, the capacitor voltage of the voltage doubler $v_{c}$ and the DC current $i_{DC}$ for all four topologies when excited by a train of bipolar pulses with amplitude $V_{s}=4\;V$, T=40 ms, and a pulse repetition period of 1 s. As expected, due to the action of the converter controller and the op-amp amplifier, the DC voltage remains constant for *FBV* and *VDV*, while it exhibits a pulse-shaped waveform for *FBR* and *VDR*. The DC current waveforms for *FBR* and *VDR* exhibit a slight knee at the beginning of each pulse because the SPV1050 integrated converter initially operates as a charge pump until a predefined threshold is reached, after which it transitions to boost-converter mode. This transient behavior is specific to the experimental implementation and is not included in the analytical model, which represents the DC/DC converter through its equivalent constant-voltage source or constant-input resistance. As observed in Figure 8, the discrepancy associated with this transient is limited to the initial portion of the pulse and occurs at relatively low current levels. Its contribution to the extracted energy is small, resulting in a difference of less than 0.6% between the measured energy and the energy obtained when this transient contribution is neglected. Finally, the capacitor voltage waveforms in Figure 8 confirm that complete discharge occurs before each pulse in *VDR*, whereas significant residual charge remains in *VDV*, consistent with the theoretical analysis.

The four topologies were tested under four pulse conditions: $V_{s}=2\;V$ or $V_{s}=4\;V$, and T=20 ms or T=40 ms. The diode voltage drop $V_{D}$ used in the analytical predictions was determined from the diode datasheet based on the expected current range. With this value of $V_{D}$, the ratio $V_{s}/V_{D}$ was approximately 9 for $V_{s}=2\;V$ and 18 for $V_{s}=4\;V$. Furthermore, the values of T=20 ms and T=40 ms resulted in $CR_{s}/T$ ratios of approximately 1 and 2, respectively. For the topologies with constant-input-voltage converters (*FBV* and *VDV*), the reference DC voltage was varied for each pulse type. For the topologies with constant-input-resistance converters (*FBR* and *VDR*), the input resistance of the converter was adjusted through resistor $R_{i}$ of the amplifier.

**Figure 9:**
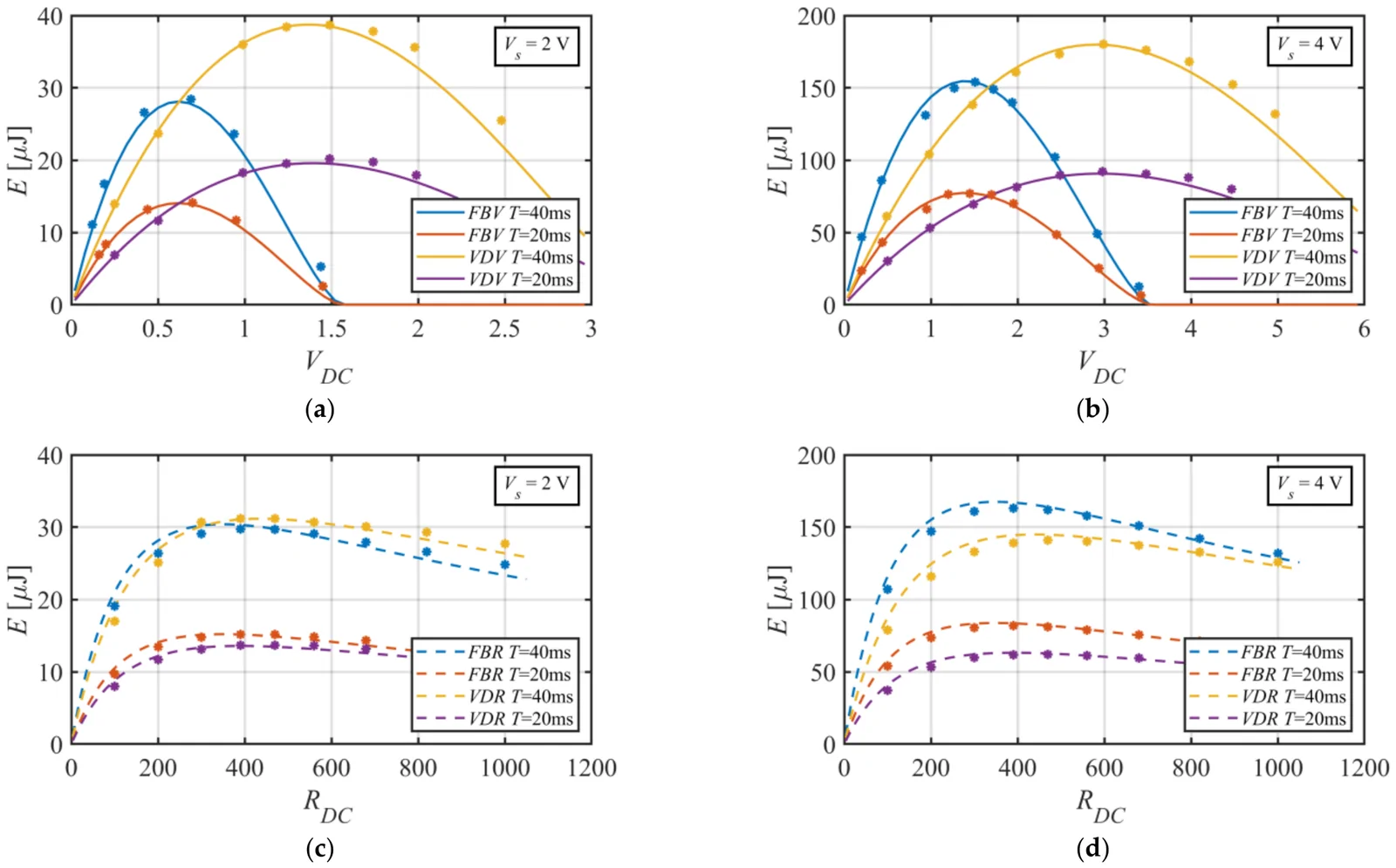
Comparison between experimental measurements (dots) and analytical predictions (lines) for the four considered interfaces: (**a**) *FBV* and *VDV* for $V_{s}=2\;V$, T=20 and 40 ms. (**b**) *FBV* and *VDV* for $V_{s}=4\;V$, T=20 and 40 ms. (**c**) *FBR* and *VDR* for $V_{s}=2\;V$, T=20 and 40 ms. (**d**) *FBR* and *VDR* for $V_{s}=4\;V$, T=20 and 40 ms.

To measure the extracted energy for the different configurations, the DC voltage waveform $V_{DC}$ and the voltage drop across the sensing resistor $R_{m}$, amplified by a factor of 10, were acquired over 10 periods using a Teledyne LeCroy HDO6104B oscilloscope. The DC current waveform $i_{DC}$ was then obtained from the voltage across $R_{m}$, accounting for the amplification factor. The instantaneous power was calculated by multiplying the $V_{DC}$ and $i_{DC}$ waveforms, and the extracted energy per pulse was obtained by integrating the instantaneous power over the 10-period acquisition interval and dividing the result by 10. The measured extracted energies are shown in Figure 9. Each plot shows the results for two values of T, namely, 20 ms and 40 ms. Figure 9a,b report the results for the *FBV* and *VDV* topologies for $V_{s}=2\;V$ and $V_{s}=4\;V$, respectively, whereas Figure 9c,d report the results for the *FBR* and *VDR* topologies for the same two source amplitudes. Each plot also includes the theoretical predictions obtained from the closed-form expressions (7), (11), (25), and (33).

For each of the 16 circuit configurations tested experimentally, the measured data shown in Figure 9 were interpolated to identify the point of maximum energy extraction. Table 2 lists the absolute percentage errors between the experimental values and those predicted by the closed-form expressions at the points of maximum energy extraction. In all 16 tested configurations, the error remains below 4%. The mean absolute percentage error is 1.44%, while the maximum error is 3.76%. Therefore, under the tested conditions, the comparison between the measured data and the analytical curves shows good agreement at the maximum-energy points, which are the points of greatest interest for applications. Minor discrepancies occur only at large values of $V_{DC}$ or $R_{DC}$, due to the simplifying assumption of constant diode voltage drop. However, this does not limit the usefulness of the models, whose purpose is to provide insight into circuit behavior and support design.

With reference to the Taylor approximations in the analytical developments, in the considered cases, the relative error in the prediction of $V_{cM}$ due to the approximation in (16) is below 0.1%. The relative error in $t_{4}$ and $E_{VDV}$ due to the approximation in (23) is below 3.5% and 2.2%, respectively. The relative error in $t_{6}$ and $E_{VDR}$ due to the approximation in (29) is below 0.5% and 0.1%, respectively.

Let us now compare the maximum energies extractable by the four interfaces. By comparing Figure 9a, obtained for $V_{s}=2\;V$, and Figure 9b, obtained for $V_{s}=4\;V$, it is clear that the optimal values of $V_{DC}$ change significantly with source pulse amplitude, in agreement with the theoretical predictions. By contrast, comparison of Figure 9c, obtained for $V_{s}=2\;V$, with Figure 9d, obtained for $V_{s}=4\;V$, confirms that the optimal $R_{DC}$ values remain unchanged when $V_{s}$ changes.

Moreover, let us consider the maximum energies extracted by the four interfaces for $V_{s}=2\;V$ and T=40 ms, reported in Figure 9a,c. The *VDV* topology extracts the highest energy, although it requires continuous MPPT, with its associated power consumption and potential risks of incorrect operation. As predicted by theory and shown in Figure 6a for $V_{s}/V_{D}\approx 9$, $CR_{s}/T\approx 1$, *VDR* and *FBR* extract comparable energy, making them viable MPPT-free alternatives. In the case of $V_{s}=4\;V$ and T=40 ms, reported in Figure 9b,d, *VDV* again extracts the highest energy, followed by *FBR*, which extracts substantially more energy than *VDR*, in agreement with the theoretical prediction in Figure 6a for $V_{s}/V_{D}\approx 18$, $CR_{s}/T\approx 1$. Overall, the experimental results confirm that the choice between *FBR* and *VDR*, both MPPT-free, depends on the value of $V_{s}/V_{D}$.

## 5. Conclusions

Closed-form expressions have been derived for the energy extracted by double-stage interfaces connected to impulsive electromagnetic energy harvesters. The usefulness of these expressions in providing insight into circuit behavior and supporting the design process has been demonstrated. The derived expressions show that a voltage doubler followed by a constant-input-voltage DC/DC converter enables higher energy extraction than the other configurations over broad ranges of the investigated parameter space. However, this configuration requires continuous MPPT operation, which may introduce additional losses and potential risks of incorrect operation. As an alternative, the *FBR* or *VDR* interfaces avoid the need for continuous MPPT, while the choice between them depends on the source-pulse characteristics and the specific rectifier implementation. Experimental measurements performed on prototypes assembled using off-the-shelf components show good agreement with the theoretical predictions.

## Figures and Tables

**Table 1:** Comparison with previous analytical studies on the analysis and design of double-stage interfaces for energy harvesters.

Reference	Waveform of the Source	Energy Harvester	Rectifier	Load of the Rectifier	Assumptions of Analysis	Type of Analysis
[39]	Continuous sinusoid	Resonant electromagnetic	Full-bridge	Resistor and capacitor	Constant voltage drop diode model	Theoretical and numerical
[47]	Continuous sinusoid	Radio Frequency	Full-bridge	Transmission line	Ideal diode model	Theoretical and numerical
[40]	Continuous sinusoid	Non-resonant electromagnetic	Full-bridge	Resistor and capacitor	Constant capacitor voltage	Theoretical and experimental
[42]	Continuous sinusoid	Piezoelectric	Voltage doubler	Resistor	Ideal diode model. Infinite resistance.	Theoretical and experimental
[44]	Continuous sinusoid	Radio Frequency	Voltage doubler	Resistor	Constant capacitor voltage	Theoretical and numerical
[45]	Continuous sinusoid	Radio Frequency	N-stage voltage multiplier	Resistor and capacitor	Constant capacitor voltage	Theoretical and experimental
This work	Bipolar pulse	Non-resonant electromagnetic	Full-bridge and voltage doubler	Constant input resistance or voltage converter	Constant voltage drop diode model	Theoretical and experimental

**Table 2:** Absolute percentage error between the measured and predicted extracted energies at the maximum-energy points of the 16 tested configurations.

			Full-Bridge Rectifier (*T* = 40 ms)	Full-Bridge Rectifier (*T* = 20 ms)	Voltage Doubler (*T* = 40 ms)	Voltage Doubler (*T* = 20 ms)
**Constant-input-voltage converter**	$V_{s}=2\;V$	**Data in** **Figure 9a**	2.14	1.19	0.07	3.76
**Constant-input-voltage converter**	$V_{s}=4\;V$	**Data in** **Figure 9b**	0.19	0.18	0.39	1.61
**Constant-input-resistance converter**	$V_{s}=2\;V$	**Data in** **Figure 9c**	1.31	0.96	1.43	1.35
**Constant-input-resistance converter**	$V_{s}=4\;V$	**Data in** **Figure 9d**	2.50	1.92	2.61	1.35

## Data Availability

The raw data supporting the conclusions of this article will be made available by the author upon request.
